# Do Sustainable Palliative Single Fraction Radiotherapy Practices Proliferate or Perish 2 Years after a Knowledge Translation Campaign?

**DOI:** 10.3390/curroncol29070404

**Published:** 2022-07-19

**Authors:** Shaheer Shahhat, Nikesh Hanumanthappa, Youn Tae Chung, James Beck, Rashmi Koul, Bashir Bashir, Andrew Cooke, Arbind Dubey, Jim Butler, Maged Nashed, William Hunter, Aldrich D. Ong, Shrinivas Rathod, Kim Tran, Julian O. Kim

**Affiliations:** 1 Undergraduate Medical Education, Max Rady College of Medicine, University of Manitoba, Winnipeg, MB R3E 3P5, Canada; sshahhat@cancercare.mb.ca; 2 Department of Radiation Oncology, Kokilaben Dhirubhai Ambani Hospital, Mumbai 400053, India; nikesh.ind@gmail.com; 3 Department of Emergency Medicine, Faculty of Medicine, University of British Columbia, Vancouver, BC V6T 1Z3, Canada; chungyt@myumanitoba.ca; 4 Department of Medical Physics, CancerCare Manitoba, Winnipeg, MB R3E 0V9, Canada; jbeck@cancercare.mb.ca; 5 Radiation Oncology, Department of Radiology, Max Rady College of Medicine, University of Manitoba, Winnipeg, MB R3E 3P5, Canada; rkoul@cancercare.mb.ca (R.K.); bbashir1@cancercare.mb.ca (B.B.); acooke1@cancercare.mb.ca (A.C.); adubey@cancercare.mb.ca (A.D.); jbutler4@cancercare.mb.ca (J.B.); mnashed1@cancercare.mb.ca (M.N.); whunter@cancercare.mb.ca (W.H.); aong3@cancercare.mb.ca (A.D.O.); srathod@cancercare.mb.ca (S.R.); 6 Radiation Oncology, Western Manitoba Cancer Center, Brandon, MB R7A 2B3, Canada; 7 Canadian Partnership Against Cancer, Toronto, ON M5H 1J8, Canada; kim.tran@partnershipagainstcancer.ca; 8 CancerCare Manitoba Research Institute, Winnipeg, MB R3E 0V9, Canada

**Keywords:** knowledge translation, palliative radiotherapy, bone metastasis, compliance fatigue

## Abstract

In early 2017, the Canadian Partnership Against Cancer and CancerCare Manitoba undertook a comprehensive knowledge translation (KT) campaign to improve the utilization of single fraction radiotherapy (SFRT) over multiple fraction radiotherapy (MFRT) for palliative management of bone metastases. The campaign significantly increased short-term SFRT utilization. We assess the time-dependent effects of KT-derived SFRT utilization 12–24 months removed from the KT campaign in a Provincial Cancer Program. This study identified patients receiving palliative radiotherapy for bone metastases in Manitoba in the 2018 calendar year using the provincial radiotherapy database. The proportion of patients treated with SFRT in 2018 was compared to 2017. Logistic regression analyses identified risk factors associated with MFRT receipt. In 2018, 1008 patients received palliative radiotherapy for bone metastasis, of which 63.3% received SFRT, a small overall increase in SFRT use over 2017 (59.1%). However, 41.1% of ROs demonstrated year-over-year decreases in SFRT utilization, indicative of a time-dependent loss of SFRT prescription habits derived from KT. Although SFRT use increased slightly overall in 2018, evidence of compliance fatigue was observed, suggestive of a time-perishing property of RO prescription behaviours derived from KT methodologies. Verification of the study’s findings in larger cohorts would be beneficial. These findings highlight the need for additional longitudinal KT reinforcement practices in the years following KT campaigns.

## 1. Background

There is significant evidence from high-quality published randomized clinical trials that single fraction radiotherapy (SFRT) is a more appropriate dose-fractionation choice when compared to multiple fraction radiotherapy (MFRT) for the palliative management of painful, uncomplicated bone metastases [1]. Several advantages of SFRT over MFRT include: non-inferior analgesic effects and post-treatment quality-of-life [1,2,3,4], non-inferior toxicity profiles [3,5], greater logistical convenience and less treatment-associated out-of-pocket expenses for patients [6,7], lessened resource impacts to healthcare systems on account of lower costs [7,8,9], and less linear accelerator time and radiotherapist workload per patient. For these reasons, SFRT is recommended over MFRT for treatment of uncomplicated bone metastases by many respected clinical guideline groups [10,11,12]. Despite its advantages, SFRT remains clinically underutilized for the management of patients with bone metastases worldwide [13,14,15]. The reasons for this knowledge-to-action gap between evidence-based recommendations for SFRT and real-world underutilization of SFRT are numerous and include unfounded fears of inadequate analgesic effect and increased toxicity with SFRT, lack of understanding of the published literature, and physician remunerative factors in some jurisdictions [13,14].

Knowledge translation (KT) campaigns serve to mitigate recognized knowledge-to-action gaps which exist between published evidence and guidelines with clinician decisions [16]. Tran et al. determined previously that a high proportion of patients in Manitoba with bone metastases were treated with MFRT (68.8%) rather than SFRT (31.2%) [15]. This finding inspired the development of a KT pilot project jointly championed by the Canadian Partnership Against Cancer and CancerCare Manitoba (CCMB; a Canadian provincial cancer program) which was designed to encourage improvements in SFRT utilization in the day-to-day practice of radiation oncologists (ROs) province wide. The KT campaign was carried out in early 2017 and was built around the Choosing Wisely Canada (CWC) [17] national campaign recommendation in support of the use of SFRT over MFRT [18].

The KT campaign pilot project and the interventions which it employed have been described previously in the report of the 2017 calendar year [19]. Briefly, the KT campaign consisted of the following elements: (1) educational outreach visits/grand rounds with external subject matter experts who reviewed the evidence and guidelines in support of SFRT utilization for painful bone metastases; (2) consensus meetings to review all of the pertinent evidence employing a cooperative Socratic style of dialogue in which questions were asked of the group members with respect to their opinions on the evidence, allowing members to make their own conclusions on the data; (3) follow-up surveys to measure intent of adopting guidelines; (4) data collection, analysis of SFRT utilization which was then presented to participating ROs in an anonymous manner at the group level. The KT campaign was based on the CWC recommendation: “Don’t recommend more than a single fraction of palliative radiation for an uncomplicated painful bone metastasis” [18]. In accordance with Cheon et al., an uncomplicated bone metastasis was defined as a painful bone metastasis unassociated with impending or existing pathologic fracture, spinal cord compression, or cauda equina compression [20]. The KT campaign was carried out in early 2017.

The impact of the KT campaign during the calendar year following the intervention (2017) were immediate and pervasive whereby every single radiation oncologist in the province increased their proportion SFRT utilization when compared to the pre-intervention period (2016). In the 12 months following the KT campaign, the provincial utilization of SFRT increased from 38.0% (2016) to 59.1% (2017) for all bone metastases, and 46.4% (2016) to 67.7% (2017) for uncomplicated bone metastases, representing year-over-year increases of 21.1% and 21.3%, respectively [19]. No further active KT interventions were mandated after the conclusion of the KT pilot project in 2017.

The purpose of this study was to assess the impact of the KT campaign beyond its immediate impact period of 12 months to the period spanning 12–24 months post-intervention and to determine if there was any aspect of time-dependent loss of KT-derived RO SFRT prescribing behaviour. Studies in the literature assessing KT interventions for effectiveness typically only extend for a short follow-up after the intervention [21,22]. Another jurisdiction has previously reported that their KT interventions for ROs resulted in only a transient increase in SFRT utilization in the first four years post-intervention and declined over the next four years almost to pre-intervention baseline [23]. Therefore, we aimed to continue to quantify SFRT use in Manitoba in 2018 and assess the long-term sustainability of KT interventions in the radiation oncology milieu.

This study aimed to determine if KT-derived RO prescription behaviour for SFRT utilization declined in the long term (during the second year removed from the KT intervention), following the KT intervention. Specifically, we sought to determine if during 2018 there was any changes in the utilization of SFRT over the reported 2017 levels (59.1% for all bone metastases; 67.7% for uncomplicated bone metastases). We also sought to identify risk factors associated with receipt of MFRT during the same time period.

## 2. Methods

CCMB is the provincially mandated and publicly funded sole provider of RT services for the Canadian Province of Manitoba, with a catchment population of approximately 1.4 million persons.

### 2.1. Data Sources and Data Extraction

All courses of palliative RT for a bone metastasis in Manitoba during the study period (1 January 2018 to 31 December 2018) were identified using the CCMB radiotherapy database. This prospectively maintained electronic administrative database is populated with variables inputted into the RT treatment directive completed by a prescribing RO prior to the initiation of any RT-related treatment procedures. The following variables were extracted from this database for each course of RT: primary tumor type (ICD-10 diagnostic code), patient sex, patient age at time of RT, RT dose, RT fractionation, and prescribing RO. The remaining characteristics were extracted from the CCMB electronic medical records including anatomic treatment site, Eastern Cooperative Oncology Group (ECOG) performance status, radiotherapy (RT) treatment intent (post-operative vs. palliative; post-operative intent RT was defined as RT within 60 days after any orthopedic surgery intervention (e.g., open reduction, internal fixation surgery), and Charlson Comorbidity Index. Diagnostic imaging reports and the electronic medical records were used to classify each bone metastasis as complicated or uncomplicated by determining the presence or report of a fracture in the targeted bony structure, spinal cord compression, or cauda equina compression. Patients were excluded from analysis if the site receiving RT was predominantly a soft tissue metastasis where the bone metastasis was only a minor component of the target volume (defined as a bone metastasis that composes <20% of the target volume as determined using each patient’s CT simulation scan). Patients treated with stereotactic body radiotherapy (SBRT) for bone metastases during 2018 were excluded from analysis since dose fractionation choices for SBRT patients are independent of the CWC guidelines.

### 2.2. Statistics

Baseline characteristics were tabulated for the entire cohort and by fractionation schedule (SFRT vs. MFRT). Differences in distribution of baseline characteristics by fractionation schedule were assessed using standard statistical tests (chi-squared, student t-test). The proportion of SFRT courses in 2018 was compared to the proportion of SFRT courses in 2017 using the one-sample z-test test for proportions. The proportion of SFRT courses prescribed by each individual RO was visualized with bar graphs for both uncomplicated and all bone metastases. The difference in proportion of bone metastases treated with SFRT were tabulated year-over-year for each individual oncologist, expressed as a percentage change. Baseline variables were assessed for potential associations for receipt of MFRT using univariable logistic regression analysis. A multivariable logistic regression model (Model 1) was built using the 2017 data employing a forward, stepwise approach. Variables with univariable associations of *p* ≤ 0.2 were considered for inclusion in the multivariable model and variables were assessed for collinearity in the model by assessing change in model variance during the forward stepwise selection process. A separate multivariable model (Model 2) was built including the data from the 2017 calendar year (previously reported) merged with the 2018 data with a variable added for year of treatment (2017 vs. 2018) in the model. The purpose of this separate model was to assess the odds ratio for receipt of MFRT by treatment year, adjusting for all the other potential confounding variables used in the 2017 logistic regression model. Multivariable associations with *p* ≤ 0.05 were considered statistically significant for this study. All analyses were conducted using STATA version 15 (Statacorp, College Station, TX, USA).

This study was conducted with the prior written approval of the University of Manitoba Health Research Ethics Board (Approval #: HS20808), and the CancerCare Manitoba Research Resource Impact Committee (Approval #: 2017-020).

## 3. Result

From 1 January 2018 to 31 December 2018, 1151 courses of palliative RT were administered to patients with a bone metastasis in Manitoba. Of these, 135 courses were excluded from the cohort because the metastasis was predominantly soft tissue metastasis with only a minor component of bony invasion. SBRT was utilized for 8 bone metastases, which were excluded from the analysis.

A total of 1008 courses of palliative RT were included in the analysis (Table 1) with a median age of 67 (range: 5–96), of whom 423 (42.0%) were women. The most common primary tumor types included: prostate (26.1%), lung (23.6%), and breast (17.3%). The most common anatomical sites of bone metastases included: skull/spine (44.6%), pelvis/proximal femur (32.3%), and upper extremity (9.2%). Retreatment to a previously irradiated site was done in 126 (12.5%) cases.

Bone metastases were classified as complicated in 319 (31.7%) cases. Amongst the whole cohort, 262 (26.0%) had fracture, 85 (8.4%) had spinal cord compression, and 30 (3.0%) had cauda equina compression. Soft tissue extension was observed in 337 (33.4%) of all bone metastases.

**Figure 1:**
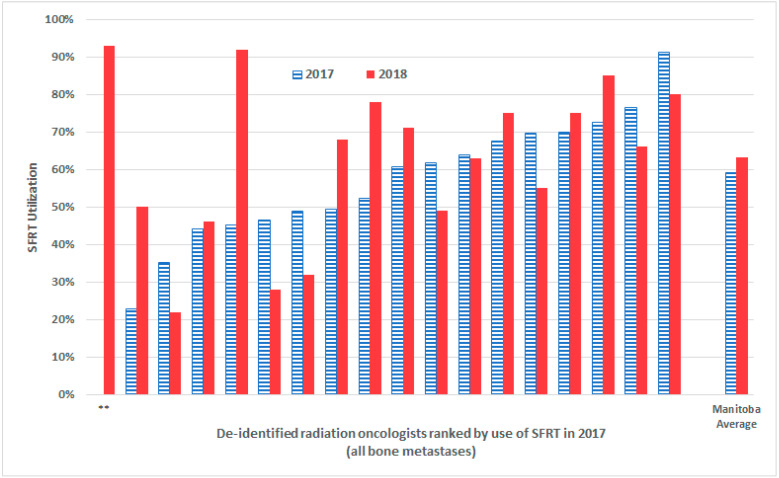
Proportion of SFRT utilized in by de-identified individual radiation oncologists for all bone metastases in 2017 and 2018 in Manitoba (**: Radiation oncologist joined the team in 2018 and has no 2017 comparison point).

During 2018, the proportion of cases treated with SFRT for all bone metastases (63.3%) significantly increased over 2017 levels of (59.1%; z-test *p* = 0.0034), representing an absolute year-over-year change of +4.2%. Among Manitoba’s seventeen ROs present during the 2017 KT campaign, nine demonstrated increased year-over-year SFRT utilization for all bone metastases in 2018, while eight demonstrated year-over-year declines in SFRT utilization (Figure 1). Year-over-year changes in SFRT utilization from 2016 (pre-KT campaign) to 2018 (2 years post campaign) for all bone metastases are tabulated in Table 2. For the group of nine ROs with increased SFRT utilization during 2018, who we will term “super adopters”, the absolute percent change in SFRT utilization in 2018 over 2017 was +17.9%, with a range of absolute percentage increases of +2.2% to +47.1%. Conversely, for the group of eight with decreased utilization during 2018 (which we will term “lapsing adopters”), the absolute percent decline in SFRT utilization in 2018 over 2017 was −9.6%, with a range of absolute percentage change of −1.4% to −18.5%. Of the eight lapsing adopters, one RO decreased their SFRT utilization to below their baseline pre-KT campaign rate in 2016, while seven have maintained rates above their pre-campaign baseline but below levels seen in the year following the KT campaign (2017).

**Figure 2:**
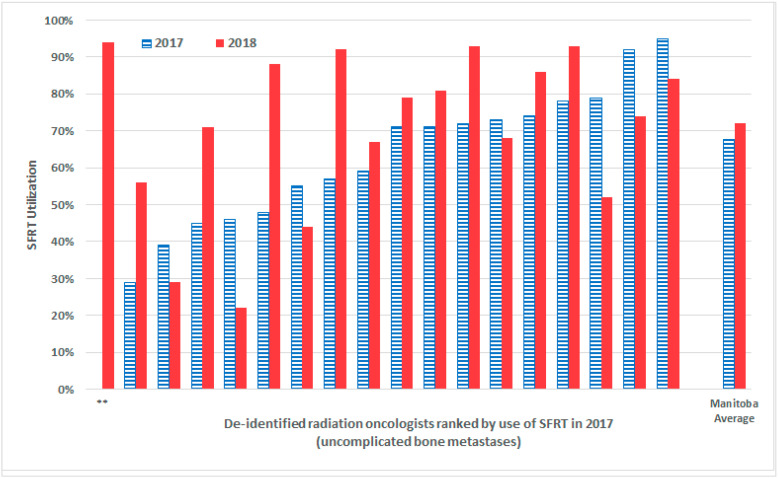
Proportion of SFRT utilized by de-identified individual radiation oncologists for uncomplicated bone metastases in 2017 and 2018 in Manitoba (**: Radiation oncologist joined the team in 2018 and has no 2017 comparison point).

When restricting the analysis to those with uncomplicated bone metastases, the proportion of cases treated with SFRT in 2018 for uncomplicated bone metastases (72.0%) significantly increased over 2017 levels (67.7%; z-test *p* < 0.00001), representing an absolute year-over-year change of +4.3%. Among Manitoba’s seventeen ROs present in the team during the 2017 calendar year, ten demonstrated increases year-over-year SFRT utilization for uncomplicated bone metastases, while seven demonstrated decreased year-over-year SFRT utilization (Figure 2). Year-over-year change in SFRT utilization from 2016 to 2018 for uncomplicated bone metastases are described in Table 3. For the group of the ten super adopters, the mean absolute percentage increase in SFRT utilization in 2018 over 2017 was +19.5%, with a range of absolute percentage increases of +7.6% to +40.8%. Conversely, for the cohort of the seven lapsing adopters, the absolute percent decline in SFRT utilization in 2018 over 2017 was −10.5%, with a range of absolute percentage declines of −5.1% to −23.6%. Of the seven lapsing adopters, one RO had decreased SFRT utilization to below their baseline pre-KT campaign rate in 2016, while the other six maintained rates above their pre-campaign baseline but below levels seen in the year following the KT campaign (2017).

In 2018, MFRT was utilized for 370 (36.7%) of all bone metastases. For patients treated with MFRT, the most common fractionation schedule prescribed were: 20 Gy in 5 (77.6%) and 30 Gy in 10 (9.7%). The proportion of complicated bone metastases were similar between 2018 (31.7%) and 2017 (32.7%). Retreatment rates were also similar year-over-year (2018: 12.5%; 2017: 13.4%).

The multivariable logistic regression analysis of the 2018 data (Table 4) revealed the following factors were significantly associated with increased odds of receiving MFRT: hematological primary malignancy (OR 3.66, 95% CI 1.90–7.05), males with non-prostate genitourinary (GU) primary malignancy (OR 2.92, 95% 1.54–5.52), other primary malignancies (includes melanomas, head and neck primaries, gynecological primaries, sarcomas, primaries of the central nervous system, and unknown primaries), OR 2.00, 95% CI 1.05–3.78), soft tissue extension (OR 3.80, 95% CI 2.68–5.40), and post-operative RT (OR 2.77, 95% CI 1.27–6.01). Odds ratios from the univariable logistic regression analyses of the 2018 data is tabulated in Table S2. 

On multivariable logistic regression of the combined 2017/2018 dataset (Table S1) including all of the covariates used in the 2017 model, patients treated in 2018 had significantly lower odds of receiving MFRT compared to patients treated in 2017 after adjusting for potential confounding variables (OR 0.70, 95% CI 0.56–0.87).

## 4. Discussion

This study found that radiation oncologist SFRT prescription behaviour imparted by KT campaigns demonstrated two important time-dependent characteristics in the second year removed from the KT campaign. Firstly, the rate of uptake in institution-wide SFRT utilization for all bone metastases has slowed from a 21.1% absolute increase in 2017 (0–12 months after the campaign) [19] to a 4.2% absolute increase in 2018 (12–24 months after the campaign; *p* = 0.0034), perhaps indicating that SFRT utilization is approaching its maximum asymptote or indicating the need for adapting our campaign message to continue reinforcing SFRT utilization. Secondly, a dichotomy has emerged whereby half of the ROs (9 of 17) who participated in the KT campaign continued to demonstrate year-over-year increases in their SFRT utilization for bone metastases in 2018, while the other half of the ROs (8 of 17) demonstrated a year-over-year decline in SFRT utilization in 2018 from their 2017 peak SFRT utilization rates. This is in contrast to the first 12 months after the campaign, when all 17 ROs who participated in the KT campaign demonstrated increased year-over-year SFRT utilization [19]. After controlling for potential confounding covariates included in Table S1, year of treatment (2018 versus 2017) remained as a statistically significant variable (*p* = 0.001). Thus, the positive impact of the KT campaign has not only decreased in momentum in the second year period post completion of the KT campaign but has also been carried by a smaller subgroup of ROs and some ROs have lapsed into old MFRT prescribing habits.

The decline in SFRT utilization observed in some of the ROs in our cohort two years post KT intervention may mirror the findings of a study conducted in British Columbia, Canada. In their jurisdiction. They examined SFRT utilization rate changes following a KT campaign in British Columbia, the authors also noted that SFRT use trended downwards after the initial uptick associated with their KT intervention [24]. The exact reason for these observed declines in SFRT utilization two years removed from the KT intervention both in the case of British Columbia and Manitoba are unknown but may be explained, in part, by several observations in the literature. Time-dependent loss of utilization of guideline compliant behaviour derived from KT campaigns has been observed in other KT milieus. In an observational audit study tracking hand hygiene compliance after a hand hygiene KT campaign in intensive care units, hand hygiene compliance increased immediately after the campaign, then subsequently declined during a two-year follow-up period as fewer intensive care units maintained strong compliance while other units returned to baseline lower compliance [25]. In another observational audit study tracking a hand hygiene KT campaign, initial hand hygiene compliance rose over the one-year period after starting the campaign, then decreased after the initial uptick [26]. These examples of time-dependent loss of KT derived behaviours suggest that the KT message compliance decays over time for healthcare practitioners and our findings suggest that radiation oncologists are not immune to forgetting lessons learned via KT campaigns. It is therefore a reasonable hypothesis that that re-exposing radiation oncologists to periodic KT refreshers may be helpful. It is also possible that lapsing radiation oncologists were overexposed to KT interventions leading to tuning out of the KT messaging, a phenomenon known as “messaging fatigue” or “campaign fatigue”. Observations in the literature suggest that healthcare professionals who are regularly overexposed to KT campaigns are not immune to messaging fatigue [27]. Exposure of healthcare workers to excessive KT messaging has been associated with information overload and mental fatigue resulting in reduced ability to distinguish important messages from irrelevant ones [28,29] which in turn can lead to suboptimal care decisions and clinician behaviour [27]. In a randomized control study in Washington, the recall of a certain public health message sent to healthcare providers was inversely proportional to the mean number of messages received per week, and the odds of recall decreased with the increase of public health messages per week [30] suggesting that KT messaging faded into “background noise”. Interestingly, that study was conducted during the H1N1 Influenza pandemic, which was associated with a dramatic increase in public health messages sent to healthcare providers, and recall rates improved as the overall message load on practitioners decreased to pre-pandemic levels [30]. More recently, evidence in the literature is emerging for the role of messaging fatigue in reducing participants’ uptake of important healthcare information during the COVID-19 pandemic [31]. This corollary lesson from the H1N1 Influenza pandemic suggests that messaging fatigue is a potential outcome of overexposure to KT interventions, and one that administrators would need to keep in mind when determining how many interventions to expose radiation oncologists to with respect to SFRT use.

Although our study demonstrated the presence of a group of super adopters and another group of lapsing adopters of SFRT for bone metastases, this division into two subgroups of ROs did not exist in retrospect prior to the 2018 results. Neither subgroup, when analyzed retrospectively (data not shown), has consistently outperformed or underperformed the other subgroup in terms of SFRT utilization. Effective KT requires early addressal of barriers to knowledge adoption [16]. It is possible that the group of lapsing adopters may have encountered new and unique barriers to knowledge adoption pertaining to SFRT during year 2 of follow-up which may not have been addressed in the original intervention. Keeping these two subgroups in mind for future analyses of SFRT utilization in Manitoba may prove useful to identify and analyze barriers to resistance to the KT campaign messaging and improve upon the gains already achieved. Often, the drivers of maintenance behaviour in KT are different from the drivers of initiation [32], and successful campaigns must recognize this difference and adapt to a changing local context to remain relevant and sustainable over time [33]. 

Although the KT campaign’s original goal was reaching an institution-level target of SFRT utilization for 60% of all bone metastases, as suggested by Tiwana et al. in a similar study in British Columbia [34], the results of this study suggest that this target may need to be revisited. Although the 2018 institution-level SFRT utilization for all bone metastases is 63.3%, we observed that only 72% of uncomplicated bone metastases received SFRT in 2018. Thus, there remains a considerable subgroup of uncomplicated bone metastases (approximately 20% of the whole cohort) for whom the SFRT guideline compliance can be improved. Moreover, there is growing evidence that SFRT is a clinically acceptable option to MFRT for subsets of patients with complicated bone metastases, specifically those with spinal cord compressions [35]. Thus, we judge that under ideal circumstances, the maximal proportion of all bone metastases which could and should be treated with SFRT in a guideline and evidence-complaint manner is approximately 80%. Our study identified several clinical subgroups of patients with lower proportions of SFRT compared to the rest of the population of patients with bone metastases, namely patients with RT prescribed post-operatively, hematological cancer primaries, non-prostate GU cancer primaries, and other cancer primaries (melanomas, head and neck primaries, gynecological primaries, sarcomas, primaries of the central nervous system, and unknown primaries). Future KT efforts will have to reaffirm the concept that SFRT for uncomplicated bone metastases is recommended for all clinical subgroups of patients including many patients from these aforementioned groups. To this end, we intend to continue to monitor compliance with SFRT utilization through audit and ad hoc feedback in an effort to keep the KT campaign message sustainable and relevant.

There are several limitations to this study. Firstly, the retrospective nature of our study cannot directly assess causality between the KT campaign and the observed effects on SFRT utilization. To mitigate this limitation, the combined 2017/2018 multivariable logistic regression analysis was built to assess the impact of treatment year on the outcome of receipt of SFRT while adjusting for many other potentially confounding variables in the model. Secondly, the decision regarding if a bone metastasis was complicated or uncomplicated prior to treatment was left to the discretion of individual ROs (i.e., it was not centrally reviewed or controlled), and thus may have had implications on the choice of SFRT vs. MFRT for individual ROs that was not captured when applying the definition of complicated employed in this study. Thirdly, since data regarding postoperative palliative RT was only collected in 2018, and not in 2017, we are unable to determine if any changes in proportion of patients treated postoperatively acted as a confounder in the year-over-year utilization of SFRT; however, this risk is expected to be minimal since postoperative RT consisted of only 5% of all bone metastases treated in 2018. Finally, although the scale of the original KT project was the full complement of ROs serving a catchment population of 1.4 million persons, this study was conducted on a total of 18 ROs. Thus, the specific proportion of super adopters and lapsing adopters seen in this study may differ if the study was repeated on a distinct population of ROs. For these reasons, further validation of our findings would be welcomed.

## 5. Conclusions

The rate of increase of SFRT utilization in Manitoba 2 years post KT intervention decreased compared to the immediate post KT-time period, and a significant proportion of ROs lapsed to lower SFRT utilization levels. Our findings suggest that KT-derived RO SFRT prescribing behaviour is time-perishing in nature. Further reinforcement of KT messaging and continued SFRT utilization audits are therefore warranted.

## Figures and Tables

**Table 1:** Baseline Characteristics of overall cohort and by fractionation schedule in 2018 (SFRT vs. MFRT) (GU: Genitourinary; ECOG: Eastern Cooperative Oncology Group).

Variable		Whole Cohort (*n* = 1008)	SFRT (*n* = 638)	MFRT (*n* = 370)	*p*-Value
Patient Characteristics					
Age (Median, Range)		67 (5–96)	69 (5–96)	65 (5–93)	0.0008
Charlson Comorbidity Index	0	540 (53.6)	325 (50.9)	215 (58.1)	0.034
	1	215 (21.3)	139 (21.8)	76 (20.5)	
	2	139 (13.8)	89 (14.0)	50 (13.5)	
	≥3	114 (11.3)	85 (13.3)	29 (7.8)	
Gender	Female	423 (42.0)	257 (40.3)	166 (44.9)	0.155
	Male	585 (58.0)	381 (59.7)	204 (55.1)	
ECOG Performance Status	0–1	475 (47.1)	294 (46.1)	181 (48.9)	0.193
	2	253 (25.1)	172 (27.0)	81 (21.9)	
	3–4	235 (23.3)	148 (23.2)	87 (23.5)	
	Unknown	45 (4.5)	24 (3.8)	21 (5.7)	
Disease Characteristics					
Tumour Type	Prostate	263 (26.1)	205 (32.1)	58 (15.7)	<0.0001
	Breast	174 (17.3)	107 (16.8)	67 (18.1)	
	Lung	238 (23.6)	150 (23.5)	88 (23.8)	
	Hematological	82 (8.1)	40 (6.3)	42 (11.4)	
	Non-prostate GU	88 (8.7)	39 (6.1)	49 (13.2)	
	Gastrointestinal	75 (7.4)	43 (6.7)	32 (8.7)	
	Other	88 (8.7)	54 (8.5)	34 (9.2)	
Site of Radiotherapy	Skull and spine	450 (44.6)	234 (36.7)	216 (58.4)	<0.0001
	Upper Extremity	93 (9.2)	77 (12.1)	16 (4.3)	
	Chest (including ribs)	68 (6.8)	48 (7.5)	20 (5.4)	
	Pelvis and proximal femur	326 (32.3)	229 (35.9)	97 (26.2)	
	Lower extremity	71 (7.0)	50 (7.8)	21 (5.7)	
Complicated Bone Metastasis	No	689 (68.4)	496 (77.7)	193 (52.2)	<0.0001
	Yes	319 (31.7)	142 (22.3)	177 (47.8)	
Fracture	No	746 (74.0)	509 (79.8)	237 (64.1)	<0.0001
	Yes	262 (26.0)	129 (20.2)	133 (36.0)	
Soft Tissue Component	No	671 (66.6)	501 (78.5)	170 (46.0)	<0.0001
	Yes	337 (33.4)	137 (21.5)	200 (54.1)	
Cord Compression	No	923 (91.6)	616 (96.6)	307 (83.0)	<0.0001
	Yes	85 (8.4)	22 (3.5)	63 (17.0)	
Cauda Equina Compression	No	978 (97.0)	633 (99.2)	345 (93.2)	<0.0001
	Yes	30 (3.0)	5 (0.8)	25 (6.8)	
Treatment Characteristics					
Retreatment	No	882 (87.5	551 (86.4)	331 (89.5)	0.152
	Yes	126 (12.5)	87 (13.6)	39 (10.5)	
Post-Operative Radiotherapy	No	958 (95.0)	618 (96.9)	340 (91.9)	<0.0001
	Yes	50 (5.0)	20 (3.1)	30 (8.1)	
Treatment Location	Winnipeg	865 (85.8)	561 (87.9)	304 (82.2)	0.011
	Brandon	143 (14.2)	77 (12.1)	66 (17.8)	
RO Years in Practice (yrs)	≤6	260 (25.8)	142 (22.3)	118 (31.9)	<0.0001
	7–16	367 (36.4)	207 (32.5)	160 (43.2)	
	≥17	381 (37.8)	289 (45.3)	92 (24.9)	

**Table 2:** Year-over-year change in proportion of bone metastases treated with SFRT by individual radiation oncologists for all bone metastases. Only clinicians who participated in the KT campaign (17/18) are included.

De-Identified Radiation Oncologist	2016 %SFRT Utilization (Pre-Campaign)	2017 %SFRT Utilization (Absolute % Change from Previous Year)	2018 %SFRT Utilization (Absolute % Change from Previous Year)
A	21%	46% (+25%)	28% (−18%)
B	25%	49% (+24%)	32% (−17%)
C	42%	70% (+28%)	55% (−15%)
D	23%	35% (+12%)	22% (−13%)
E	44%	61% (+17%)	49% (−12%)
F	77%	91% (+14%)	80% (−11%)
G	32%	76% (+44%)	66% (−10%)
H	22%	64% (+44%)	63% (−1%)
I	24%	44% (+20%)	46% (+2%)
J	34%	70% (+36%)	75% (+5%)
K	53%	68% (+15%)	75% (+7%)
L	50%	61% (+11%)	71% (+10%)
M	55%	73% (+18%)	85% (+12%)
N	34%	52% (+18%)	78% (+16%)
O	16%	49% (+33%)	68% (+19%)
P	0%	23% (+23%)	50% (+27%)
Q	23%	45% (+22%)	92% (+47%)

**Table 3:** Year-over-year change in proportion of bone metastases treated with SFRT by individual radiation oncologists for uncomplicated bone metastases. Only clinicians who participated in the KT campaign (17/18) are included.

De-Identified Radiation Oncologist	2016 %SFRT Utilization (Pre-Campaign)	2017 %SFRT Utilization (Absolute % Change from Previous Year)	2018 %SFRT Utilization (Absolute % Change from Previous Year)
A	67%	79% (+12%)	52% (−27%)
B	14%	46% (+34%)	22% (−24%)
C	38%	92% (+54%)	74% (−18%)
D	36%	55% (+19%)	44% (−11%)
E	80%	95% (+15%)	84% (−11%)
F	26%	39% (+13%)	29% (−10%)
G	26%	73% (+47%)	68% (−5%)
H	10%	59% (+49%)	67% (+8%)
I	77%	71%% (−6%)	79% (+8%)
J	46%	71% (+25%)	81% (+10%)
K	64%	74% (+10%)	86% (+12%)
L	67%	78% (+11%)	93% (+15%)
M	48%	72% (+24%)	93% (+21%)
N	24%	45% (+21%)	71% (+26%)
O	0%	29% (+29%)	56% (+27%)
P	29%	57% (+28%)	92% (+35%)
Q	43%	48% (+5%)	88% (+40%)

**Table 4:** Multivariable Logistic Regression Analysis for Receipt of MFRT in 2018 (Model 1) (GU: Genitourinary; ECOG: Eastern Cooperative Oncology Group).

Variable		Multivariable Odds Ratio (95%CI)	*p*-Value
Age (years)	5 to ≤57	Ref	Ref
	58 to ≤66	0.96 (0.61 to 1.50)	0.608
	67 to ≤75	0.84 (0.52 to 1.35)	0.519
	≥76	0.78 (0.48 to 1.27)	0.484
Sex	Female	Ref	Ref
	Male	1.13 (0.75 to 1.69)	0.558
ECOG Performance Status	0–1	Ref	Ref
	2	0.59 (0.40 to 0.87)	0.007
	3–4	0.57 (0.38 to 0.86)	0.007
Charlson Score	0	Ref	Ref
	1	0.73 (0.48 to 1.10)	0.129
	2	0.77 (0.47 to 1.24)	0.28
	≥3	0.49 (0.27 to 0.86)	0.014
Tumour Type	Prostate	Ref	Ref
	Breast	1.71 (0.86 to 3.40)	0.127
	Lung	1.63 (0.98 to 2.72)	0.06
	Hematological	3.66 (1.90 to 7.05)	<0.0001
	Non-Prostate GU	2.92 (1.54 to 5.52)	<0.0001
	Gastrointestinal	1.73 (0.88 to 3.42)	0.115
	Other	2.00 (1.05 to 3.78)	0.034
Treatment Site	Skull/Spine	Ref	Ref
	Upper Extremity	0.30 (0.16 to 0.58)	<0.0001
	Thorax	0.40 (0.20 to 0.78)	0.007
	Pelvis	0.61 (0.43 to 0.89)	0.01
	Lower Extremity	0.54 (0.27 to 1.08)	0.08
Complicated Bone Metastasis	Uncomplicated	Ref	Ref
	Complicated	1.69 (1.18 to 2.41)	0.004
Soft Tissue Extension	No	Ref	Ref
	Yes	3.80 (2.68 to 5.40)	<0.0001
Retreatment	No	Ref	Ref
	Yes	0.67 (0.41 to 1.09)	0.105
Post-Operative Radiotherapy	No	Ref	Ref
	Yes	2.77 (1.27 to 6.01)	0.01
Treatment Location	Winnipeg	Ref	Ref
	Brandon	1.30 (0.78 to 2.15)	0.308
Radiation Oncologist Years in Practice	≤6	Ref	Ref
	7 to 16	0.80 (0.51 to 1.24)	0.312
	≥17	0.30 (0.19 to 0.48)	<0.0001

## Data Availability

The datasets used during and/or analyzed during the current study are available from the corresponding author on reasonable request.

## Supplementary Materials

The following supporting information can be downloaded at: https://www.mdpi.com/article/10.3390/curroncol29070404/s1, Supplemental Table S1: Multivariable Logistic Regression Analysis (Model 2) for Receipt of MFRT merging the 2017 and 2018 datasets and includes a treatment year variable (2017 versus 2018; Supplemental Table S2: Univariable Logistic Regression Analysis for Receipt of MFRT in 2018.
