# Supplementary material for: Do Sustainable Palliative Single Fraction Radiotherapy Practices Proliferate or Perish 2 Years after a Knowledge Translation Campaign?
Source: Curr Oncol. 2022 Jul 19;29(7):5097–109. doi: 10.3390/curroncol29070404 (PMC9324375; doi:10.3390/curroncol29070404)
Supplement: Supplementary file 1 [file curroncol-29-00404-s001.zip › curroncol-1804907-supplementary.pdf]

**Table S1.** Multivariable Logistic Regression Analysis (Model 2) for Receipt of MFRT merging the 2017 and 2018 datasets and includes a treatment year variable (2017 versus 2018) (GU: Genitourinary; ECOG: Eastern Cooperative Oncology Group).

| Variable | | Multivariable Odds Ratio (95%CI) | *p*-Value |
|---|---|---|---|
| Age (years) | 5 to ≤ 58 | Ref | Ref |
| | 59 to ≤ 68 | 0.85 (0.63 to 1.14) | 0.273 |
| | 69 to ≤ 76 | 0.82 (0.59 to 1.14) | 0.246 |
| | ≥ 77 | 0.56 (0.40 to 0.79) | 0.001 |
| Sex | Female | Ref | Ref |
| | Male | 1.23 (0.93 to 1.63) | 0.142 |
| ECOG Performance Status | 0-1 | Ref | Ref |
| | 2 | 0.70 (0.54 to 0.89) | 0.004 |
| | 3-4 | 0.56 (0.42 to 0.75) | <0.0001 |
| Charlson Score | 0 | Ref | Ref |
| | 1 | 0.84 (0.64 to 1.10) | 0.203 |
| | 2 | 0.77 (0.55 to 1.07) | 0.117 |
| | ≥3 | 0.53 (0.35 to 0.79) | 0.002 |
| Tumour Type | Prostate | Ref | Ref |
| | Breast | 1.87 (1.19 to 2.96) | 0.007 |
| | Lung | 1.76 (1.22 to 2.55) | 0.003 |
| | Hematological | 3.13 (2.00 to 4.90) | <0.0001 |
| | Non-Prostate GU | 2.62 (1.69 to 4.06) | <0.0001 |
| | Gastrointestinal | 1.86 (1.18 to 2.95) | 0.008 |
| | Other | 1.99 (1.27 to 3.13) | 0.003 |
| Treatment Site | Skull/Spine | Ref | Ref |
| | Upper Extremity | 0.41 (0.26 to 0.66) | <0.0001 |
| | Thorax | 0.47 (0.31 to 0.73) | 0.001 |
| | Pelvis | 0.73 (0.56 to 0.94) | 0.014 |
| | Lower Extremity | 0.80 (0.53 to 1.22) | 0.301 |
| Fracture | No | Ref | Ref |
| | Yes | 1.12 (0.87 to 1.44) | 0.399 |
| Soft Tissue Extension | No | Ref | Ref |
| | Yes | 3.14 (2.43 to 4.05) | <0.0001 |
| Spinal Cord Compression | No | Ref | Ref |
| | Yes | 2.63 (2.34 to 5.63) | <0.0001 |
| Cauda Equina Compression | No | Ref | Ref |
| | Yes | 3.29 (1.71 to 6.33) | <0.0001 |
| Retreatment | No | Ref | Ref |
| | Yes | 0.50 (0.35 to 0.71) | <0.0001 |
| Treatment Location | Winnipeg | Ref | Ref |
| | Brandon | 1.63 (1.13 to 2.34) | 0.008 |
| Radiation Oncologist Years in Practice | ≤ 7 | Ref | Ref |
| | 8 to 17 | 0.90 (0.65 to 1.25) | 0.540 |
| | ≥18 | 0.58 (0.42 to 0.82) | 0.002 |
| Treatment Year | 2017 | Ref | Ref |
| | 2018 | 0.70 (0.56 to 0.87) | 0.001 |

**Table S2.** Univariable Logistic Regression Analysis for Receipt of MFRT in 2018 (GU: Genitourinary; ECOG: Eastern Cooperative Oncology Group).

| **Variable** | | **Univariable Odds Ratio (95% CI)** | ***p*-Value** |
|---|---|---|---|
| Age (years) | 5 to ≤ 57 | Ref | Ref |
| | 58 to ≤ 66 | 0.84 (0.58 to 1.21) | 0.339 |
| | 67 to ≤ 75 | 0.76 (0.53 to 1.09) | 0.131 |
| | ≥ 76 | 0.52 (0.36 to 0.76) | 0.001 |
| Sex | Female | Ref | Ref |
| | Male | 0.83 (0.64 to 1.07) | 0.155 |
| ECOG Performance Status | 0-1 | Ref | Ref |
| | 2 | 0.76 (0.55 to 1.06) | 0.103 |
| | 3-4 | 0.95 (0.69 to 1.32) | 0.779 |
| Charlson Score | 0 | Ref | Ref |
| | 1 | 0.83 (0.60 to 1.15) | 0.255 |
| | 2 | 0.85 (0.58 to 1.25) | 0.408 |
| | ≥3 | 0.52 (0.33 to 0.81) | 0.004 |
| Tumour Type | Prostate | Ref | Ref |
| | Breast | 2.21 (1.45 to 3.38) | <0.0001 |
| | Lung | 2.07 (1.40 to 3.07) | <0.0001 |
| | Hematological | 3.71 (2.20 to 6.25) | <0.0001 |
| | Non-Prostate GU | 4.44 (2.66 to 7.41) | <0.0001 |
| | Gastrointestinal | 2.63 (1.53 to 4.53) | <0.0001 |
| | Other | 2.23 (1.32 to 3.74) | 0.003 |
| Treatment Site | Skull/Spine | Ref | Ref |
| | Upper Extremity | 0.23 (0.13 to 0.40) | <0.0001 |
| | Thorax | 0.45 (0.26 to 0.79) | 0.005 |
| | Pelvis | 0.46 (0.34 to 0.62) | <0.0001 |
| | Lower Extremity | 0.46 (0.26 to 0.78) | 0.004 |
| Complicated Bone Metastasis | Uncomplicated | Ref | Ref |
| | Complicated | 3.20 (2.43 to 4.22) | <0.0001 |
| Fracture | No | Ref | Ref |
| | Yes | 2.21 (1.66 to 2.95) | <0.0001 |
| Soft Tissue Extension | No | Ref | Ref |
| | Yes | 4.30 (3.26 to 5.68) | <0.0001 |
| Spinal Cord Compression | No | Ref | Ref |
| | Yes | 5.75 (3.47 to 9.51) | <0.0001 |
| Cauda Equina Compression | No | Ref | Ref |
| | Yes | 9.17 (3.48 to 24.18) | 0.121 |
| Retreatment | No | Ref | Ref |
| | Yes | 0.75 (0.50 to 1.11) | 0.153 |
| Post-Operative Radiotherapy | No | Ref | Ref |
| | Yes | 2.73 (1.52 to 4.87) | 0.001 |
| Treatment Location | Winnipeg | Ref | Ref |
| | Brandon | 1.58 (1.11 to 2.26) | 0.012 |
| Radiation Oncologist Years in Practice | ≤ 6 | Ref | Ref |
| | 7 to 16 | 0.93 (0.68 to 1.28) | 0.657 |
| | ≥17 | 0.38 (0.27 to 0.54) | <0.0001 |
